# High-throughput sequencing for the study of bacterial pathogen biology

**DOI:** 10.1016/j.mib.2014.06.002

**Published:** 2014-06

**Authors:** Paul R McAdam, Emily J Richardson, J Ross Fitzgerald

**Affiliations:** The Roslin Institute and Edinburgh Infectious Diseases, University of Edinburgh, Easter Bush Campus, Edinburgh EH25 9RG, United Kingdom

## Abstract

A revolution in sequencing technologies in recent years has led to dramatically increased throughput and reduced cost of bacterial genome sequencing. An increasing number of applications of the new technologies are providing broad insights into bacterial evolution, epidemiology, and pathogenesis. For example, the capacity to sequence large numbers of bacterial isolates is enabling high resolution phylogenetic analyses of bacterial populations leading to greatly enhanced understanding of the emergence, adaptation, and transmission of pathogenic clones. In addition, RNA-seq offers improved quantification and resolution for transcriptomic analysis, and the combination of high-throughput sequencing with transposon mutagenesis is a powerful approach for the identification of bacterial determinants required for survival *in vivo*. In this concise review we provide selected examples of how high throughput sequencing is being applied to understand the biology of bacterial pathogens, and discuss future technological advances likely to have a profound impact on the field.

## Highlights

• High throughput sequencing (HTS) is revolutionizing research into bacterial pathogens.
• HTS reveals that bacterial pathogens may undergo considerable diversification during infection.
• HTS can allow tracing of outbreak origin and transmission.
• HTS offers advantages over existing transcriptomic technologies for understanding global gene expression in bacteria.
• Transposon mutagenesis and HTS is a powerful combination for identifying bacterial determinants required for *in vivo* survival.

## Graphical abstract

**Figure:**
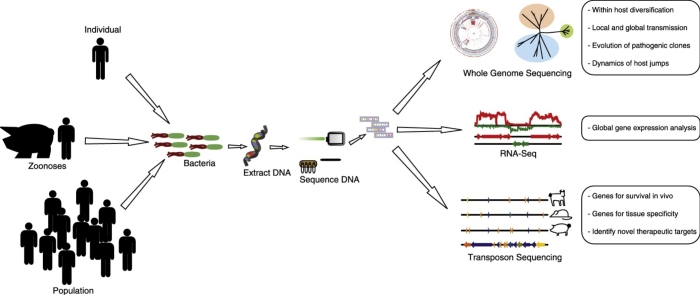

**Current Opinion in Microbiology** 2014, **19**:106–113

This review comes from a themed issue on **Novel technologies in microbiology**

Edited by **Emmanuelle Charpentier** and **Luciano Marraffini**

For a complete overview see the Issue and the Editorial

Available online 14th July 2014

**http://dx.doi.org/10.1016/j.mib.2014.06.002**

1369-5274/© 2014 The Authors. Published by Elsevier Ltd. This is an open access article under the CC BY license (http://creativecommons.org/licenses/by/3.0/).

## Introduction

The development of new technologies enabling rapid, inexpensive, and high-throughput DNA sequencing (HTS) that offer clear advantages over traditional Sanger sequencing has revolutionized the field of bacterial genomics [1•, 2]. Furthermore, the recent development of high-throughput ‘benchtop’ sequencers is empowering laboratories to sequence their bacteria of interest independently of specialist sequencing centres [2, 3]. An array of different technologies have been developed with the common feature that they parallelize the sequencing process, leading to the production of thousands or millions of sequence reads concurrently (for a review of the HTS technologies see [1•, 2]). HTS is being applied in a myriad of ways to address fundamental questions concerning the biology of infectious diseases. The high resolution offered by HTS allows the inference of transmission pathways during global pandemics and localized outbreaks, identification of molecular mechanisms underpinning the emergence of pathogenic clones, and the evolutionary analysis of bacterial populations during infection of individual patients. HTS also provides the potential for transcriptomic analyses with advantages over traditional hybridization approaches, including genome-wide coverage, accurate quantification, and single nucleotide resolution (for recent comprehensive reviews, see [4, 5•]). In addition, the combination of HTS with transposon mutagenesis leading to the development of approaches such as Tn-seq [6], transposon-directed insertion site sequencing (TraDIS) [7^•^], insertion sequencing (INseq) [8], and high-throughput insertion tracking by deep sequencing (HITS) [9] has facilitated the screening of libraries of hundreds of thousands of bacterial mutants to identify determinants required for survival during growth *in vivo* or in other specific growth conditions (for recent comprehensive reviews see [10, 11]).

In the current concise review, we will summarize selected recent studies that have applied HTS to answer important questions regarding the success of major bacterial pathogens. We provide an overview of some of the insights which can be derived from the application of these techniques.

## Study of bacterial evolution during infection

The progression and outcome of infectious disease is determined by the dynamics of host–pathogen interactions, and recent studies employing HTS have offered novel insights into the evolution of bacterial pathogens during the course of colonization and infection [12•, 13, 14••, 15]. For example, an emerging theme in infectious disease research is the extent of genetic and phenotypic diversification that may occur among the infecting bacterial population within an individual host. In particular, *Pseudomonas aeruginosa*, *Staphylococcus aureus*, *Mycobacterium abscessus*, *Mycobacterium tuberculosis*, and *Burkholderia dolosa* have been demonstrated to undergo considerable diversification during infection, resulting in ‘clouds of diversity’ that originated from a single or closely related group of infecting bacteria [12•, 14••, 15, 16, 17•, 18]. During infection, random advantageous mutations may become fixed within sub-populations, due to selective pressures such as co-infection with other microorganisms, the host immune response, and antimicrobial chemotherapy [12•, 14••, 15, 17•, 18, 19]. Of note, cystic fibrosis (CF) patients are at particular risk of pulmonary infections, and a number of studies have utilized HTS to examine the genetic diversification of bacterial populations during long-term infection of CF patients [12•, 15, 18, 20]. For example, convergent evolution represented by independent mutations affecting O-antigen switching has been identified in chronic *B. dolosa* infections [15]. Furthermore, mutations influencing the smooth to rough morphotype transition of *M. abscessus* spp. during infection of the CF lung have been identified [20, 21], and distinct polymorphisms of loci influencing the alternative sigma factor (SigB) of *S. aureus* were identified in multiple sub-lineages of *S. aureus* within a single CF patient [18]. The study also revealed mutations underlying antibiotic resistance which occurred during infection as demonstrated in other studies of *S. aureus* chronic infection [22, 23]. Loss of virulence factor production has also been described during therapeutic *Escherichia coli* colonization of human patients with recurrent urinary tract infections [24^••^]. Numerous mutations associated with reduced virulence and adaptation to oxidative stresses, and the recurrence of mutations within individual patients strongly suggested that host-specific selective pressures influence microevolution during infection [24^••^]. In addition, a long term *S. aureus* carriage study identified an overall pattern of purifying selection within asymptomatically colonized hosts [13]. An enrichment of premature stop codons was observed in invasive bloodstream isolates when compared to carriage isolates from an individual who developed a fatal bacteraemia, implying that specific genetic changes in carriage isolates may be functionally important in pathogenesis [14^••^].

**Figure 1:**
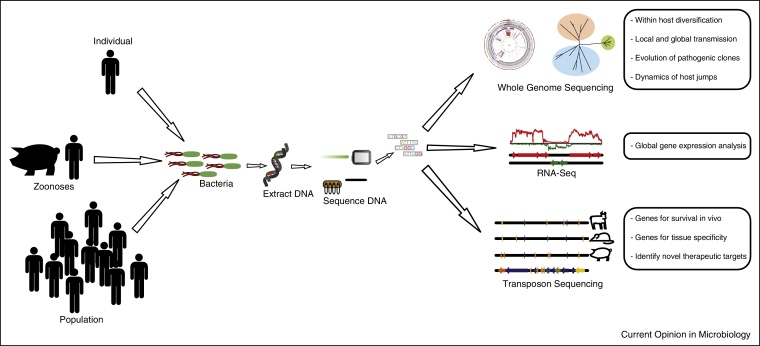
Schematic diagram summarizing the applications of high throughput sequencing for studies of the epidemiology, evolution and pathogenesis of bacterial infections.

Differences in the composition of resident bacterial populations between healthy and disease states are increasingly being described, and deep sequencing meta-genomic methods can capture greater diversity from the microbiota in comparison to traditional methods relying on PCR amplification and Sanger sequencing [25]. For example, decreased microbial diversity in CF patients in comparison to healthy controls is associated with more severe inflammation [26] and distinct shifts in metabolic pathways have been identified [27]. Additionally, the effects of antimicrobial therapies on the gut microbiota have been investigated revealing increased phage mobilization [28, 29], and profound shifts in composition that persist after the cessation of therapy [30] (Figure 1).

## Identification of bacterial determinants required for pathogenesis

The application of HTS for measuring the bacterial transcriptome (RNA-seq) in different environmental conditions has considerable advantages over traditional hybridization-based techniques. The method requires isolation of RNA followed by reverse transcription to cDNA to allow library construction before HTS. Specifically, the inclusion of strain-specific genetic material, single base-pair resolution, and more accurate quantification of relative levels of gene expression are improvements on previous approaches using microarrays. Although RNA-seq technology has been relatively slow to be employed for bacterial transcriptomic studies due in part to technical challenges of contaminating rRNA, an increasing number of studies are now being published. For example, comparative gene expression analysis of the opportunistic human pathogen *Aggregatibacter actinomycetemcomitans in vivo* and in a biofilm model *in vitro* revealed differential expression of 14% of the transcriptome, providing new information relevant to the metabolic pathways important for infection [31]. Other selected studies have shown differential expression of master regulatory genes in *Streptococcus pneumoniae* [6], genes essential for the survival of *Haemophilus influenzae* outside of the host [32], and survival of *Salmonella* Typhi in bile [7^•^]. In addition, RNA-seq identified the extent of the *S.* Typhi *ompR* regulon [33] and revealed that the *E. coli* plasmid pO157_sal regulates the expression of chromosomal genes associated with the stress response, antibiotic resistance, and virulence [34].

A particularly powerful application is the combination of HTS with transposon mutagenesis. Briefly, libraries made up of bacteria each with single transposon insertions into non-essential genes are used as an innoculum for experimental infections or for culture in defined conditions. Subsequently, input and output populations are subjected to HTS allowing the relative quantification of mutants in each population. Accordingly, the complement of genes required for survival can be identified. For example, the application of transposon-directed insertion-site (TraDIS) sequencing to *Salmonella enterica* serovars *S.* Typhi and *S.* Typhimurium revealed a conserved core of 281 genes required for growth in both serovars [35]. Attenuation of O-antigen genes and ferric [Fe(III)] genes was observed in *S.* Typhimurium and *S.* Typhi respectively [35], which may partly reflect the distinct host tropisms of each serovar. A Tn-seq study of adaptation of *S. pneumoniae* to the nasopharynx and the lung indicated an array of genes associated with response to hydrogen peroxide which were important during lung infection, while genes associated with response to temperature and sucrose were involved in survival in the nasopharynx [36^••^]. In contrast, a study of the interactions of *Moraxella catarrhalis* with the host respiratory epithelia reported no difference in expression of adhesion factors between adhesive and planktonic states [37].

Finally, in a novel application of Tn-seq, the dynamics of *H. influenzae* and influenza A virus co-infection were examined in a mouse model of respiratory infection. Of note, *H. influenzae* genes associated with oxidative stress were required during co-infection with influenza A compared to *H. influenzae* infection alone [38^•^]. The use of HTS-transposon approaches is revealing much about the mechanisms of bacterial survival *in vivo* and in doing so may indicate novel therapeutic targets for controlling infection.

## Identifying the source and transmission routes of disease outbreaks

Traditional bacterial typing methods are often of limited use for epidemiological investigations of infectious disease outbreaks due to their low level of discriminatory power [39]. The comparison of whole genome sequences can provide the ultimate level of nucleotide resolution between bacterial isolates and accordingly has the potential to identify transmission events within and between hospitals and in the community [3, 17•, 40•, 41, 42, 43•, 44••, 45, 46, 47]. Importantly, the application of rapid sequencing of suspected outbreak strains can inform both clinical practice and infection control procedures [17^•^]. The level of genetic variation observed among strains epidemiologically linked to an outbreak can help determine whether a point source or multiple strains are responsible [3, 40•, 44••, 48]. For example, based on multiple locus tandem repeats typing analysis, a clonal population of *M. tuberculosis* was implicated as the cause of a sustained outbreak of tuberculosis in Canada. However, in a seminal study by Gardy *et al.*, integration of genome sequence information and social network analysis led to the discovery that the outbreak was caused by two circulating lineages sustained by crack cocaine users [44^••^]. In addition, HTS has been applied retrospectively to analyse *S. aureus* epidemics in hospitals revealing the dynamics of outbreaks confined to single hospital wards [3, 40•], and transmission between the hospital and community settings [17^•^]. The directionality of transmission, although often ambiguous, may sometimes be inferred by combining epidemiological data and analysis of bacterial population genome sequences [12•, 15]. For example, in a study of *Burkholderia dolosa* associated with chronic infections in a CF patient cohort, transmission events could be inferred from the phylogeny based on patterns of shared polymorphisms [15]. Also, a study of patients with *M. abscessus* subspecies *massiliense* pulmonary infections showed greater genetic diversity among isolates from a single patient than from different patients, suggestive of inter-patient transmission events [12^•^]. Recently, the development of novel bioinformatic algorithms has facilitated the identification of transmission events while accounting for the heterogeneity present in infecting bacterial populations [49].

## Understanding the molecular basis for the emergence of pathogenic clones

The capacity to sequence large numbers of closely related isolates allows high-resolution phylogenies to be reconstructed, which may provide insights into the processes underlying the emergence and spread of pathogenic clones. Bacterial populations accumulate random mutations over time through inherent mutation rates specific for the organism and its ecology. Estimates of the mutation rate for a given bacterial population allow the construction of time-calibrated phylogenetic trees [50]. In particular, the application of Bayesian phylogenetic methods to sequences of closely related bacteria, can allow a time-scaled reconstruction of their evolutionary history and geographic dissemination, and may also reveal genetic events that correlate with the emergence of successful clones. The evolutionary history of an increasing array of bacterial pathogens has been examined using this approach including *S. aureus*, *Shigella sonnei, S.* Typhimurium and *Vibrio cholerae* [42, 43•, 51, 52, 53, 54, 55, 56]. In a concise review of this nature we can provide just a few recent examples. Methicillin-resistant *S. aureus* (MRSA) is an important cause of nosocomial infection in the United Kingdom, and recent studies have described genetic events preceding the emergence of the major hospital-adapted lineages EMRSA-15 and EMRSA-16, including polymorphisms associated with increased antimicrobial resistance and reduced virulence [42, 43•]. The reduction in virulence likely represents a fitness compensation in response to the increased energy costs associated with antibiotic resistance. In addition, a recent HTS study of invasive *S.* Typhimurium circulating in sub-Saharan Africa revealed the existence of two distinct epidemics that correlated strongly with peaks in HIV incidence in the region, suggesting that the increased number of immunocompromised hosts in sub-Saharan Africa has been a major contributor to the success of invasive *S.* Typhimurium [53]. Similarly, a study of *S.* Typhimurium DT104 from livestock and human hosts employed Bayesian phylogenetic methods with extended analysis of host-tropism using Markov jump methods to demonstrate that circulating DT104 strains represent independent epidemics. The data indicated that there had been limited cross-species transmission, contradicting a long-standing assumption regarding the zoonotic spread of *S.* Typhimurium [54].

In another HTS-based evolutionary study, it was demonstrated that the 7th cholera pandemic comprised of three independent waves, with the latter two being driven through acquisition of the SXT antibiotic resistance element by *V. cholerae*. This resulted in resistance to the commonly used anti-cholera therapies [52]. In the aftermath of the 2010 earthquake in Haiti, the country suffered from a prolonged outbreak of cholera. Phylogenetic reconstruction based on whole genome sequences of *V. cholerae* from affected patients identified several intercontinental transmission events originating from a source population in the Bay of Bengal [52]. Further analysis revealed that Haitian epidemic strains are related to strains circulating in Nepal, implicating movements of Nepali peacekeepers in the emergence of the epidemic [55] following a single introduction to Haiti [56].

The impact of human clinical interventions on the emergence of successful bacterial clones has been demonstrated for the *S. pneumoniae* PMEN1 lineage [57, 58]. Following introduction of a conjugate polysaccharide vaccine, capsule switching was observed [57] leading to the emergence of the vaccine escape serotype 19A. It was revealed that the geographic distribution of serotype 19A has rapidly expanded to replace vaccine-susceptible serotypes [58]. Further work based on HTS of 616 *S. pneumoniae* isolates identified variation in recombination rates between branches of the species’ phylogeny and also revealed an association between specific genetic determinants of *S. pneumoniae* and the age of the host. These data are consistent with adaptation of *S. pneumoniae* in response to the maturing host immune response [59^••^].

HTS also played a central role in the near real-time characterization of a high mortality outbreak of haemolytic-uraemic syndrome (HUS) in central Europe during 2011 [60, 61, 62], rapidly revealing that the increased virulence of the O104:H4 outbreak was likely due to acquisition of a Shiga-toxin encoding prophage [62], and that the strain was refractory to antibiotic therapy due to carriage of an extended spectrum β-lactamase [60].

## The molecular basis of bacterial host switches

The high resolution phylogenetic analysis possible using HTS has enhanced our understanding of the capacity of bacterial pathogens to switch host species and adapt to survive and spread among novel host populations [51, 63, 64, 65, 66]. In particular, several studies have identified livestock as reservoirs for emerging bacterial strains capable of causing disease in humans [51, 63, 65]. For example, Price *et al.* examined the multi-host association of the ST398 clone of *S. aureus* by HTS providing evidence for the emergence of antibiotic resistant strains resulting from the use of antibiotics in the livestock industry [63]. Similarly, Spoor *et al.* demonstrated that cows are a potential reservoir of new strains of *S. aureus* with the capacity for pandemic spread in humans [51].

In addition to understanding the dynamics of cross-species transmission, HTS allows high-resolution analysis of genetic correlates of host specificity. For example, in one of the few genome-wide association studies (GWAS) of bacteria to be carried out, Shepphard *et al.* identified a genomic region encoding vitamin B_5_ biosynthesis components which is associated with adaptation of *Campylobacter jejuni* to the bovine host [67^•^]. Also, a *S.* Typhimurium ST4/74 transposon mutant pool was used to infect avian, bovine, and porcine hosts revealing host-specific gene repertoires, including genes associated with anaerobic growth in the avian host [68^••^]. Finally, HTS of isolates of the host-restricted DT2 lineage of *S.* Typhimurium and closely related strains suggested that adaptation of the DT2 lineage to the rock pigeon was not mediated by acquisition of novel genetic material, but rather by polymorphisms in the pre-existing genetic repertoire [69].

## Future applications of HTS for understanding the biology of bacterial pathogens

The pace of development of sequencing technologies shows no sign of slowing, and platforms capable of single-molecule sequencing and ever increasing read lengths offer the possibility of highly accurate assemblies of individual pathogens within a microbial community [70]. While bacterial culture has previously been an essential step for the isolation of enough genomic DNA for whole genome sequencing, novel culture-free methodologies offer the ability to sequence un-culturable organisms. Such applications have relevance for investigating the cause of infectious diseases of unknown aetiology. Furthermore, the rapid diagnosis and *in silico* determination of sensitivity profiles of pathogens without the necessity for culture have obvious benefits for the treatment of clinical infections [71•, 72]. Furthermore, transcriptomic analysis of complex populations of bacteria within the microbiota will be theoretically feasible. To date, transcriptomic studies have focused on either the pathogen or the host. However, a comprehensive understanding of host–pathogen interactions would require simultaneous analysis of gene expression of both parties during infection. Dual RNA-seq offers the potential for transcriptomic analysis of both host and pathogen during the course of colonization and infection but there are technical difficulties to overcome before routine implementation of this technology is feasible, including removal of both bacterial and host rRNA, and large scale differences in relative amounts of RNA for host and pathogen [73]. Finally, the availability of genome sequences for large numbers of well-defined clinical isolates lends itself to the application of GWAS to bacteria, a method originally developed for human genetic association analysis which has the potential for unbiased identification of genetic determinants associated with a given phenotype [74]. For example molecular correlates of virulence or host-specificity may be determined through simultaneous analysis of genome data and the results from virulence assays [67•, 75, 76].

Overall the development of HTS technologies has revolutionized how we approach fundamental research into infectious diseases. Without doubt the new approaches will result in a very enhanced understanding of the biology of bacterial pathogens which will ultimately lead to improved infection control.

## References and recommended reading

Papers of particular interest, published within the period of review, have been highlighted as:

• of special interest
•• of outstanding interest
